# Anti-Hypertensive Medications and Cardiovascular Events in Older Adults with Multiple Chronic Conditions

**DOI:** 10.1371/journal.pone.0090733

**Published:** 2014-03-10

**Authors:** Mary E. Tinetti, Ling Han, Gail J. McAvay, David S. H. Lee, Peter Peduzzi, John A. Dodson, Cary P. Gross, Bingqing Zhou, Haiqun Lin

**Affiliations:** 1 Department of Medicine, Yale School of Medicine, New Haven, Connecticut, United States of America; 2 Yale School of Public Health, New Haven, Connecticut, United States of America; 3 Oregon State University/Oregon Health and Science University, College of Pharmacy, Portland, Oregon, United States of America; 4 Department of Medicine, Brigham and Women's Hospital/Harvard Medical School, Boston, Massachusetts, United States of America; INRCA, Italy

## Abstract

**Importance:**

Randomized trials of anti-hypertensive treatment demonstrating reduced risk of cardiovascular events in older adults included participants with less comorbidity than clinical populations. Whether these results generalize to all older adults, most of whom have multiple chronic conditions, is uncertain.

**Objective:**

To determine the association between anti-hypertensive medications and CV events and mortality in a nationally representative population of older adults.

**Design:**

Competing risk analysis with propensity score adjustment and matching in the Medicare Current Beneficiary Survey cohort over three-year follow-up through 2010.

**Participants and Setting:**

4,961 community-living participants with hypertension.

**Exposure:**

Anti-hypertensive medication intensity, based on standardized daily dose for each anti-hypertensive medication class participants used.

**Main Outcomes and Measures:**

Cardiovascular events (myocardial infarction, unstable angina, cardiac revascularization, stroke, and hospitalizations for heart failure) and mortality.

**Results:**

Of 4,961 participants, 14.1% received no anti-hypertensives; 54.6% received moderate, and 31.3% received high, anti-hypertensive intensity. During follow-up, 1,247 participants (25.1%) experienced cardiovascular events; 837 participants (16.9%) died. Of deaths, 430 (51.4%) occurred in participants who experienced cardiovascular events during follow-up. In the propensity score adjusted cohort, after adjusting for propensity score and other covariates, neither moderate (adjusted hazard ratio, 1.08 [95% CI, 0.89–1.32]) nor high (1.16 [0.94–1.43]) anti-hypertensive intensity was associated with experiencing cardiovascular events. The hazard ratio for death among all participants was 0.79 [0.65–0.97] in the moderate, and 0.72 [0.58–0.91] in the high intensity groups compared with those receiving no anti-hypertensives. Among participants who experienced cardiovascular events, the hazard ratio for death was 0.65 [0.48–0.87] and 0.58 [0.42–0.80] in the moderate and high intensity groups, respectively. Results were similar in the propensity score-matched subcohort.

**Conclusions and Relevance:**

In this nationally representative cohort of older adults, anti-hypertensive treatment was associated with reduced mortality but not cardiovascular events. Whether RCT results generalize to older adults with multiple chronic conditions remains uncertain.

## Introduction

Cardiovascular (CV) events such as myocardial infarction (MI) and stroke are common in older adults [1], [2]. Blood pressure control is central to cardiovascular risk reduction [3]–[5]. Evidence from randomized controlled trials (RCTs) demonstrate the beneficial effect of treatment on the risk of CV events in even very elderly adults with hypertension [6]–[9]. A recent Cochrane review reported a relative risk reduction of 28% with treatment of hypertension in older adults [6]. The absolute reduction in CV events over a mean of 4.5 years was from 15.3 to 11 events per 100 participants. There was modest benefit for total mortality, although not for persons over age 80 years [6].

Participants in RCTs of anti-hypertensive medications are not representative of older adults seen in clinical practice [6]–[10]. The Hypertension in the Very Elderly Trial (HYVET), for example, enrolled only individuals in good cognitive and physical health with few chronic conditions; most participants were from Eastern Europe and China [7], [8]. Older individuals with multiple chronic conditions in the U.S. may not experience the same effects of anti-hypertensive medications as participants in RCTs. Previous RCTs assembled cohorts either not on treatment or whose medications were discontinued prior to enrollment. Over 80% of older adults with hypertension receive anti-hypertensive medications [11]. The clinical question for older adults, therefore, is more often not whether to start but rather whether there is benefit to continuing anti-hypertensive medications.

The effect of anti-hypertensive medications on CV events and mortality in representative samples of older adults, most of whom have received anti-hypertensive treatment for many years and have coexisting conditions, remains largely unexplored. The aim of the current study was to estimate the association between intensity of anti-hypertensive medications and CV events and mortality in a nationally representative population of older adults.

## Methods

### Study Design and Sample

The study sample included Medicare Current Beneficiary Survey (MCBS) participants enrolled from 2004–2007 and followed through 2010. MCBS is a nationally representative sample of Medicare beneficiaries obtained using stratified multi-stage sampling from the Centers for Medicare and Medicaid Services (CMS) enrollment file [12]. Eligibility for the current study included age over 70 years; community-living at baseline; inpatient, physician or outpatient claim for hypertension during the first year of enrollment in MCBS; and participation in fee-for-service Medicare. Medicare Advantage beneficiaries were excluded because they lack health claims. Of the 5,124 MCBS members who met these criteria, the 4,961(96.8%) with medication data available constituted the study cohort. Follow-up was up to three years, until death, enrollment in Medicare Advantage, or the end of the study. The study was deemed exempt from review by the Yale University Human Investigation Committee because it involved existing, publically-available, de-identified data.

### Descriptive Data

Chronic conditions were ascertained from Medicare, hospital, outpatient, and physician claims data. The Elixhauser comorbidity scale was computed based on the International Classification of Diseases (ICD)-9 codes [13]. Socio-demographic, behavioral, and functional data were obtained from the Access to Care baseline interviews [12]. Depression was defined by a claim for depression or self-reported depression plus loss of interest. Cognitive impairment or dementia was considered present if there was a claim for dementia or cognitive disorder or self-reported memory loss, plus either trouble concentrating or difficulty making decisions that interfered with activities of daily living (ADLs).

### Medication Use Data

Prescription medications were ascertained by direct observation during in-person interviews. The anti-hypertensive medication classes included diuretics, renin angiotensin system (RAS) blockers (angiotensin-converting-enzyme inhibitors and angiotensin receptor blockers), beta-blockers, calcium channel-blockers, centrally-acting anti-adrenergic agents, and other (e.g. peripheral acting anti-adrenergic agents; vasodilators) [14]. Combination medications were included in each relevant class. Each participant's daily dose of each anti-hypertensive medication received was converted to a standardized daily dose based on the corresponding defined daily dose (DDD) proposed the World Health Organization International Working Group for Drug Statistics Methodology [15]. The daily anti-hypertensive medication exposure intensity (abbreviated as anti-hypertensive intensity) for each participant was derived by dividing the total DDD units across all anti-hypertensive medications by the number of days under observation. We also calculated the number of anti-hypertensive medication classes (0, 1, 2, and 3+) each participant used.

### Outcomes

The primary outcomes included cardiovascular (CV) events and total mortality. The composite CV event outcome, ascertained by Medicare inpatient claims during years two through four included coronary events, strokes, and hospitalizations for heart failure (ICD9 disease code 428); Coronary events included acute coronary syndrome (MI or unstable angina) (ICD9 disease codes 410 or 411); ICD9 procedure codes for coronary bypass (3610–3616), revascularization (3619, 362, 3631–3634, 3639), angioplasty (3603), stent placement (3606,3607), or coronary artery thrombolytic infusion (3604); or physician Current Procedural Technology (CPT) codes for coronary artery bypass (33508, 33510–33519, 33521–33523, 33533–33536) or revascularization (33140, 33141). Stroke included ICD9 disease codes for cerebral hemorrhage (430, 431, 432.0, 432.1, 432.9), occlusion and stenosis with or without mention of cerebral infarction (433.01, 433.11, 433.21, 433.31, 433.81, 433.91, 434.90; 434.91), thrombosis (434.01), embolism (434.10, 434.11) or physician CPT) code for cerebral thrombolysis (37195) [16]. Deaths were ascertained through the Vital Status file.

### Statistical analysis

Baseline characteristics were summarized using means and standard deviations or frequencies and percentages. Anti-hypertensive intensity was trichotomized, based on the data distribution and clinical judgment, as none (0 to <0.2 DDD units), moderate (0.2–2.5 DDD units), and high (>2.5 DDD units).

To address potential confounding by indication, we estimated a propensity score (PS) using a cumulative logit regression model, with the 3-level anti-hypertensive intensity as an ordinal outcome [17]–[19]. Propensity score is an estimate of the probability that individuals would receive treatment. This method is used in nonrandomized studies to account for differences between treated and untreated individuals based on “propensity” or likelihood to be treated. The PS model included 36 participant characteristics (shown in Table 1) associated with the likelihood of being prescribed anti-hypertensive medications, including factors associated with underlying cardiovascular risk and overall health and functioning. We examined the distribution of the derived PS and checked the balance of each covariate across the three anti-hypertensive intensity groups using a cumulative logit model, adjusting for PS as a continuous covariate [18]–[20].

**Table 1:** Baseline Characteristics by Antihypertensive Medication Intensity^a^.

	Full Cohort (N = 4,961)				Propensity Score-Matched Cohort (N = 2,849)				
Characteristics^b^	None (N = 697)	Moderate (N = 2,711)	High (N = 1,553)	P-value (P-Value)^c^	None (N = 662)	Moderate (N = 1,455)	High (N = 732)	Standardized Difference^d^d1 (d2)	
Age, mean (SD), Years	80.1 (5.9)	80.4 (5.8)	79.8 (5.6)	.037 (.712)	80.1 (5.9)	80.4 (5.8)	80.2 (5.6)	.072	(.057)
Female sex	385 (55.2)	1657 (61.1)	1008 (64.9)	<.001 (.883)	370 (55.9)	848 (58.3)	444 (60.7)	.046	(.074)
Non-white race	66 (9.5)	284 (10.5)	228 (14.7)	<.001 (.398)	61 (9.2)	118 (8.1)	90 (12.3)	.034	(.080)
Hispanic ethnicity	42 (6.0)	128 (4.7)	71 (4.6)	.225 (.795)	39 (5.9)	72 (4.9)	36 (4.9)	.031	(.027)
Education < high school	463 (66.4)	1871 (69.0)	1080 (69.5)	.210 (.802)	437 (66.0)	1024 (70.4)	478 (65.3)	.096	(.038)
Perceived health less than very good	353 (50.6)	1577 (58.2)	995 (64.1)	<.001 (.431)	338 (51.1)	764 (52.5)	401 (54.8)	.034	(.055)
Current smoker	60 (8.6)	186 (6.9)	86 (5.5)	.007 (.824)	53 (8.0)	111 (7.6)	47 (6.4)	.018	(.044)
BMI ≥30	88 (12.6)	514 (19.0)	425 (27.4)	<.001 (.107)	86 (13.0)	195 (13.4)	140 (19.1)	.011	(.113)
Prescription drug insurance	517 (74.2)	1883 (69.5)	1100 (70.8)	.415 (.859)	491 (74.2)	1035 (71.1)	528 (72.1)	.070	(.020)
Blood pressure measured within 6 months	651 (93.4)	2552 (94.1)	1493 (96.1)	.002 (.947)	621 (93.8)	1351 (92.9)	691 (94.4)	.058	(.009)
Dependent in any BADLs	237 (34.0)	1040 (38.4)	642 (41.3)	.001 (.458)	224 (33.8)	503 (34.6)	241 (32.9)	.055	(.010)
Dependent in any IADLs	355 (50.9)	1516 (55.9)	920 (59.2)	<.001 (.480)	338 (51.1)	744 (51.1)	384 (52.5)	.020	(.009)
Mobility difficulty	308 (44.2)	1326 (48.9)	839 (54.0)	<.001 (.389)	292 (44.1)	644 (44.3)	319 (43.6)	.019	(.016)
Health limits social Activities	258 (37.0)	999 (36.8)	593 (38.2)	.452 (.545)	239 (36.1)	512 (35.2)	240 (32.8)	.021	(.028)
Urinary Incontinence	112 (16.1)	514 (19.0)	313 (20.2)	.036 (.609)	108 (16.3)	256 (17.6)	118 (16.1)	.029	(.001)
Cognitive Impairment	153 (22.0)	495 (18.3)	224 (14.4)	<.001 (.762)	136 (20.5)	233 (16.0)	87 (11.9)	.021	(.143)
Depression	128 (18.4)	447 (16.5)	238 (15.3)	.079 (.856)	113 (17.1)	221 (15.2)	103 (14.1)	.011	(.048)
Fall injury in past year	67 (9.6)	273 (10.1)	128 (8.2)	.118 (.967)	60 (9.1)	141 (9.7)	59 (8.1)	.011	(.009)
Uses assistive device	117 (16.8)	558 (20.6)	346 (22.3)	.006 (.477)	111 (16.8)	277 (19.0)	124 (16.9)	.086	(.002)
Osteoporosis	188 (27.0)	661 (24.4)	338 (21.8)	.005 (.787)	178 (26.9)	379 (26.0)	187 (25.5)	.011	(.012)
Prior MI	12 (1.7)	65 (2.4)	31 (2.0)	.967 (.914)	12 (1.8)	32 (2.2)	11 (1.5)	.020	(.010)
Heart failure	150 (21.5)	754 (27.8)	532 (34.3)	<.001 (.253)	147 (22.2)	329 (22.6)	173 (23.6)	.011	(.011)
Diabetes	215 (30.8)	923 (34.0)	698 (44.9)	<.001 (.241)	208 (31.4)	402 (27.6)	245 (33.5)	.095	(.001)
Diabetes (Complicated)	71 (10.2)	342 (12.6)	274 (17.6)	<.001 (.297)	69 (10.4)	134 (9.2)	91 (12.4)	.053	(.053)
Prior stroke	101 (14.5)	410 (15.1)	265 (17.1)	.064 (.842)	98 (14.8)	202 (13.9)	102 (13.9)	.019	(.034)
Cardiac arrhythmia	250 (35.9)	1108 (40.9)	630 (40.6)	.135 (.709)	231 (34.9)	565 (38.8)	266 (36.3)	.073	(.024)
Valvular disease	181 (26.0)	771 (28.4)	483 (31.1)	.009 (.698)	169 (25.5)	371 (25.5)	201 (27.5)	.021	(.015)
Atrial fibrillation	126 (18.1)	604 (22.3)	372 (24.0)	.005 (.723)	118 (17.8)	294 (20.2)	133 (18.2)	.037	(.003)
ESRD	73 (10.5)	405 (14.9)	300 (19.3)	<.001 (.208)	71 (10.7)	183 (12.6)	98 (13.4)	.077	(.079)
Blood loss anemia	33 (4.7)	133 (4.9)	68 (4.4)	.553 (.844)	27 (4.1)	67 (4.6)	29 (4.0)	.017	(.001)
Weight loss	97 (13.9)	307 (11.3)	127 (8.2)	<.001 (.389)	79 (11.9)	167 (11.5)	66 (9.0)	.034	(.061)
PVD	206 (29.6)	847 (31.2)	487 (31.4)	.502 (.836)	194 (29.3)	436 (30.0)	212 (29.0)	.025	(.002)
Psychosis	44 (6.3)	127 (4.7)	62 (4.0)	.025 (.924)	39 (5.9)	58 (4.0)	29 (4.0)	.078	(.074)
Elixhauser Comorbidty Score ≥3	461 (66.1)	1827 (67.4)	1084 (69.8)	.051 (.315)	428 (64.7)	926 (63.6)	475 (64.9)	.017	(.033)
Statin	234 (33.6)	1153 (42.5)	755 (48.6)	<.001 (.540)	225 (34.0)	508 (34.9)	281 (38.4)	.008	(.074)
No. medications other than anti-hypertensives mean (SD)	5.6 (4.2)	6.0 (4.4)	6.8 (4.9)	<.001 (.282)	5.4 (3.7)	5.2 (3.6)	5.5 (3.7)	.021	(.016)

Abbreviations: BADL, basic activities of daily living; BMI, body mass index; COPD, chronic obstructive pulmonary disease; ESRD, end stage renal disease; IADL, instrumental activities of daily living; MI, myocardial infarction; PVD, peripheral vascular disease; RAS, renin angiotensin system; SD, standard deviation.

a Members of the 2004–2007 Medicare Current Beneficiary Survey with hypertension. Anti-hypertensive medication intensity is defined in Methods. Data are presented as number (%) unless otherwise indicated.

b All variables were included in the propensity model.

c The P-values in parentheses are the propensity score adjusted P-values.

d d1 and d2 are the standardized differences between the moderate or high anti-hypertensive intensity groups, and the no hypertensive group, respectively; d2 is in parentheses. Standardized differences compare the mean differences between the comparison groups in units of the pooled standard deviations of the comparison groups, with a value of < 0.10 denoting negligible imbalance. See Statistical Analyses for details. In the full cohort before matching), 25 of the 36 PS covariates had d1 and/or d2 greater than 0.10 in contrast to 2 of 36 covariates for the PS-matched subcohort.

To enhance the comparability of the anti-hypertensive intensity groups, we assembled a more homogeneous subcohort using a greedy matching algorithm based on the estimated PS [18], [28]. Non-users, the smallest group, was treated as the index group. A caliper width of 0.02 standard deviation of the mean PS in this group was used to match one or more participants from the moderate and high intensity groups with the non-users. The balance of covariates before and after the matching was evaluated using standardized differences (STDs) between each user group and the non-user group [18], [21]. The STD contrasts the group means of each covariate in units of the pooled standard deviations of the groups, allowing for assessment of balance of covariates across groups with different sizes. Although there is no universally adopted gold standard, a standardized difference < 0.10 is considered balanced [18].

We used proportional hazard models to examine the relationship between groups and the outcomes [18], [22]. We used standard Cox regression to analyze mortality and a competing risk model using subdistribution hazards regression to analyze CV events accounting for potential bias due to the high attrition from mortality [23]–[25]. In these analyses, deaths with no CV event anytime during follow-up were treated as the competing event. We repeated the mortality models among participants who experienced a CV event. For this analysis, we reset the time zero as the onset of the first CV event and followed these participants until death or end of follow-up.

We first fitted regression models in the full cohort with and without adjusting for a continuous propensity score and 19 *a priori* selected covariates. Hazard ratios (HR) and 95% confidence intervals (CIs) were estimated for moderate and high intensity, in reference to the no anti-hypertensive group. Model fit and the proportional hazard assumptions were checked by examining Martingale residuals and cumulative incidence plots, and by testing anti-hypertensive intensity by survival time interactions [18], [22], [24]. Analyses were repeated in the PS- matched subcohort, with the PS-matched strata treated as a clustering factor [17], [20], [21].

The standard survival analyses of total mortality in both the full cohort and the PS-matched subcohort, and the competing risk analyses of the CV outcomes in the full cohort were performed using the SAS version 9.3 (SAS Institute, Inc., Cary, NC), PHREG procedure or the SAS macro %PSHREG [24]. To account for potential non-proportional hazards, a time-averaged effect was estimated in the standard and the subdistribution hazard regression models for competing risk analyses using the same SAS macro [24], [25]. The competing risk analyses in the PS-matched subcohort were estimated using the R package crrSC, where the matching is accounted for as a clustering factor [26]. A P-value of 0.05 (two-tailed) was used to denote statistical significance.

## Results

### Participant Characteristics and Frequency of Outcomes in the Full Cohort

The mean age of participants was 80.2 (5.8) years; 3,050 (61.5%) were female. Characteristics are presented in **Table 1** for the 697 participants (14.1%) in the no anti-hypertensive medication group, the 2,711 (54.6%) in the moderate intensity group, and the 1,553 (31.3%) in the high intensity group. Participants in the three groups differed on many characteristics; none of these differences were statistically significant after adjusting for propensity score (**Table 1**). Among anti-hypertensive medication users, 28.3% took one, 35.8% took two, and 35.9% took three or more classes of anti-hypertensive medications. The frequency of anti-hypertensive use was 2,809 (56.6%) for RAS-blockers, 2,691 (54.2%) for diuretics, 2,277 (45.9%) for beta-blockers, 1,695 (34.2%) for calcium channel-blockers, and 349 (7%) for other anti-hypertensive classes.

During the three-year follow-up, 1,247 participants (25.1%) experienced cardiovascular events; 407 participants (8.2%) suffered coronary events while 270 participants (5.4%) experienced strokes. A total of 732 participants (14.8%) had at least one hospitalization for heart failure. The proportion of participants experiencing a CV event during follow-up according to anti-hypertensive intensity is shown in **Table 2**.

**Table 2:** Frequency of Cardiovascular Events and Mortality in Older Adults with Hypertension According to Anti-hypertensive Medication Intensity.

	Anti-hypertensive Medication Intensity No. (%)^a^					
	Full Cohort (N = 4961)			Propensity Score-Matched Cohort (N = 2849)		
Outcomes	None (N = 697)	Moderate (N = 2711)	High (N = 1553)	None (N = 662)	Moderate (N = 1455)	High (N = 732)
Composite CV outcome^b^	138 (19.8)	649 (23.9)	460 (29.6)	129 (19.5)	303 (20.8)	168 (22.9)
Coronary event^c^	46 (6.6)	229 (8.5)	132 (8.5)	43 (6.5)	110 (7.6)	52 (7.1)
Stroke	45 (6.5)	126 (4.7)	99 (6.4)	42 (6.3)	62 (4.3)	42 (5.7)
Hospitalization for heart failure^d^	78 (11.2)	447 (16.5)	351 (22.6)	73 (11.0)	204 (14.0)	110 (15.0)
Total mortality	135 (19.4)	459 (16.9)	243 (15.7)	123 (18.6)	228 (15.7)	104 (14.2)
Mortality in participants who experienced a primary CV event^e^	62/138 (44.9)	227/649 (35.0)	141/460 (30.7)	56/129 (43.4)	110/303 (36.3)	50/168 (29.8)

Abbreviations: CV, cardiovascular; MI, myocardial infarction.

a The number and percent of participants who experienced at least one CV event during follow-up.

b The composite CV outcome included any of coronary event, stroke, and hospitalization for heart failure during follow-up. Participants could experience more than one CV event. A total of 1247 participants (25.1%) experienced 2123 cardiovascular events. A total of 407 participants (8.2%) suffered 466 coronary events while 270 participants (5.4%) experienced 303 strokes. A total of 732 participants (14.8%) had at least one hospitalization for heart failure including 582 (11.7%) with one, 180 (3.6%) with two, and 114 (2.3%) with three or more hospitalizations.

c Coronary event included MI, unstable angina, and cardiac revascularization.

d The sample size is smaller for heart failure than the other outcomes because the prebaseline and year 1 claims were used to establish a diagnosis of heart failure; follow-up for heart failure was therefore two, rather than three, years.

e Deaths among participants who experienced a CV event (any of coronary event, stroke, or hospitalization for heart failure) anytime during follow-up. The denominator is the number of persons who experienced a CV event.

A total of 837 participants (16.9%) died during follow-up. Of these deaths, 430 (51.4%) occurred in participants who experienced CV events at some time during follow-up. The proportion of all participants who died according to anti-hypertensive group is shown in **Table 2**, as is the proportion of participants experiencing CV events who died.

### Characteristics and Frequency of Outcomes in the Propensity Score-Matched Subcohort

The PS-matched sample included 95% of the no anti-hypertensive (N = 662), 53.7% of the moderate intensity (N = 1455), and 47.1% of the high intensity (N = 732) groups. The three groups were well-matched on all characteristics except for a higher percentage of high intensity users with a BMI>30 and of nonusers with cognitive impairment (**Table 1**). The incidence of CV events, total deaths, and deaths among the subgroup that experienced CV events in the PS-matched subcohort are shown in **Table 2**.

### Relationship between Anti-hypertensive Intensity and Cardiovascular Events and Mortality

**Figure 1:**
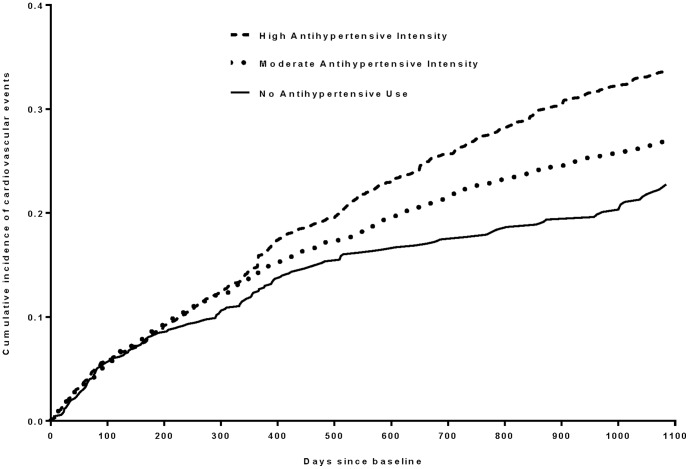
3-year cumulative incidence of cardiovascular events according to anti-hypertensive intensity in older adults with hypertension. Legend: The cumulative incidence was estimated using a subdistribution hazards regression model, with the cardiovascular events as the primary outcome and mortality among participants with no primary CV event during follow-up as the competing outcome. Follow-up period was three years. Anti-hypertensive intensity was trichotomized into no anti-hypertensive use, moderate anti-hypertensive intensity, and high anti-hypertensive intensity as defined in the Methods. The variables included in the propensity score are noted in Table 1. The model adjusted for year of study entry, propensity score as a continuous variable, age, gender, prior myocardial infarction, prior stroke, heart failure, diabetes atrial fibrillation, valvular heart disease, renal disease, current smoking status, statin use, difficulty walking, obesity, depression, cognitive impairment, number of non-antihypertensive medications, self-perceived health, blood pressure taken within past six month. Vertical line (Y Axis) represents cumulative incidence probability (%); horizontal line (X Axis) represents time in days from study entry to onset of first cardiovascular event.

The association between anti-hypertensive intensity and CV events is shown in **Table 3**. In the full cohort, after adjusting for propensity score and other covariates, neither moderate (adjusted HR 1.08 [95% CI, 0.89–1.32]) nor high (adjusted HR 1.16 [95% CI, 0.94–1.43]) intensity was associated with experiencing CV events in comparison to no anti-hypertensive use. Results were similar in the PS-matched subcohort (**Table 3**). The three-year adjusted cumulative incidence of CV events in the full cohort was 22.7% for the no anti-hypertensive group, 27.0% in the moderate intensity group, and 33.7% in the high intensity group (**Figure 1**).

**Table 3:** Effect of Antihypertensive Medication Intensity on Cardiovascular Events and Mortality in Older Adults with Hypertension.

	Hazard Ratio (95% CI)^a^			
	Full Cohort (N = 4961)		Propensity Score-Matched Cohort (N = 2849)	
	Moderate Antihypertensive Intensity Group	High Antihypertensive Intensity Group	Moderate Antihypertensive Intensity Group	High Antihypertensive Intensity Group
Composite CV outcome^b^	1.08 (0.89–1.32)	1.16 (0.94–1.43)	1.07 (0.85–1.35)	1.13 (0.88–1.44)
Coronary event	1.20 (0.86–1.67)	1.05 (0.73–1.51)	1.14 (0.78–1.68)	1.02 (0.69–1.53)
Stroke	0.72 (0.50–1.02)	0.99 (0.68–1.43)	0.70 (0.46–1.06)	1.00 (0.64–1.57)
Hospitalization for HF	1.24 (0.96–1.61)	1.43 (1.09–1.88)	1.25 (0.93–1.69)	1.29 (0.94–1.77)
Total mortality^c^	0.79 (0.65–0.97)	0.72 (0.58–0.91)	0.75 (0.60–0.93)	0.69 (0.52–0.91)
Death in those experiencing CV events during follow-up^d^	0.65 (0.48–0.87)	0.58 (0.42–0.80)	—	—

Abbreviations: CV, cardiovascular; HF, heart failure.

a Hazard ratios and 95% confidence intervals were estimated for moderate and high antihypertensive intensity group, in reference to the no anti-hypertensive group.

b The outcome was occurrence during follow-up of acute coronary syndrome (MI, unstable angina, or cardiac revascularization), stroke, or hospitalization for heart failure. The variables included in the propensity score are noted in Table 1. For the full cohort analyses, the models included year of study entry, age, gender, race, prior myocardial infarction, prior stroke, prior hospitalization for heart failure, diabetes, atrial fibrillation, valvular heart disease, renal disease, statin use, current smoking status, difficulty walking, obesity, depression, cognitive impairment, number of non-antihypertensive medications, self-perceived health, blood pressure taken within past six month) and a continuous variable for propensity score. For the PS-matched cohort analyses, the models included the same 19 covariates with the propensity score matched sets as a clustering factor.

c The outcome was death during follow-up among all cohort members. The model adjusted for the same covariates as for the cardiovascular event model.

d The outcome was death among the subgroup of participants who experienced a CV event (coronary event, stroke, or hospitalization for heart failure) any time during follow-up. See Methods for analytical details. PS-matched analyses not performed due to small sample size.

In secondary analyses, we looked at the individual categories of CV outcomes. Anti-hypertensive use was not associated with occurrence of coronary events. There was a statistically insignificant lower risk of stroke with moderate, but not high, anti-hypertensive intensity (**Table 3**). Conversely, the risk of hospitalizations for heart failure was higher among participants receiving anti-hypertensives compared with those who did not; this finding reached statistical significance only in the high intensity group in the full cohort.

The risk of death during follow-up was significantly lower in the moderate and high intensity groups compared with participants not receiving anti-hypertensives in the full cohort and PS-matched subcohort (**Table 3**). In secondary analysis, the risk of death was 35% lower [95%CI, 13–52%] among moderate, and 42% lower [20–58%] among high intensity anti-hypertensive users than nonusers among the subgroup of participants who experienced a CV event during follow-up (**Table 3**).

In analyses evaluating the number of anti-hypertensive medication classes, persons receiving three or more anti-hypertensive classes were 44% more likely to experience CV events than persons receiving no anti-hypertensives in the full cohort (**Table 4**). The comparable increased risk was 38% in the PS-matched subcohort. Similar to results for anti-hypertensive intensity, mortality decreased with increasing number of anti-hypertensive classes (**Table 4**). Other than a slightly higher risk of CV events with beta-blockers than with other anti-hypertensives, there was no difference in the association between class and CV events or mortality for any class of anti-hypertensive (**Table 4**).

**Table 4:** Effect of Number and Classes of Anti-hypertensive Medication on Cardiovascular Events and Mortality in Older Adults with Hypertension.

	Hazard Ratio (95% CI)^a^			
	Full Cohort (N = 4961)		Propensity Score-Matched Cohort (N = 2849)^d^	
No. anti-hypertensive medications classes^b^	Cardiovascular event^c^	Total mortality^c^	Cardiovascular event^c^	Total mortality^d^
1	1.00 (0.77–1.31)	0.77 (0.60–0.98)	1.01 (0.75–1.36)	0.74 (0.57–0.95)
2	1.17 (0.91–1.50)	0.65 (0.51–0.83)	1.21 (0.91–1.60)	0.62 (0.47–0.83)
3+	1.44 (1.12–1.85)	0.63 (0.49–0.80)	1.38 (1.04–1.85)	0.58 (0.83–0.78)
**Anti-hypertensive Medication Class^e^**				
Renin-angiotensin system blocker	1.13 (0.99–1.27)	0.84 (0.72–0.97)	1.02 (0.86–1.22)	0.86 (0.71–1.05)
Beta-blocker	1.27 (1.12–1.43)	0.89 (0.76–1.03)	1.28 (1.07–1.52)	0.76 (0.63–0.92)
Calcium-channel blocker	1.10 (0.97–1.24)	0.83 (0.72–0.97)	1.05 (0.87–1.26)	0.87 (0.72–1.05)
Diuretic	1.17 (1.03–1.34)	0.93 (0.79–1.08)	1.18 (0.98–1.41)	0.88 (0.72–1.10)

a Hazard ratios were estimated for users of 1, 2 and 3 or more anti-hypertensive medication classes, in reference to those who did not use anti-hypertensive medications.

b Represented in the models by three dummy indicators.

c The outcome was occurrence during follow-up of acute coronary syndrome (MI, unstable angina, or cardiac revascularization), stroke, or hospitalization for heart failure. The variables included in the propensity score are noted in Table 1. For the propensity score adjusted cohort analyses, the models adjusted for year of study entry, age, gender, race, prior myocardial infarction, prior stroke, prior hospitalization for heart failure, diabetes, atrial fibrillation, valvular heart disease, renal disease, statin use, current smoking status, difficulty walking, obesity, depression, cognitive impairment, number of non-antihypertensive medications, self-perceived health, blood pressure taken within past six month) and a continuous variable for propensity score. For the PS-matched cohort analyses, the models adjusted for the same covariates, accounting for the propensity score matched sets as a clustering factor.

d The outcome was death during follow-up among all cohort members. The models adjusted for the same covariates as for the cardiovascular event model.

e Participants may use more than one class; model hazard ratio contrasts users versus non-users (reference) of each anti-hypertensive medication class, adjusting for use of other anti-hypertensive classes. The propensity score adjusted and propensity scare matched analyses were performed as described above.

## Discussion

In this nationally representative cohort of older adults we found that anti-hypertensive treatment was associated with a reduction in mortality but not cardiovascular events. Several factors could explain the lack of effect of anti-hypertensives on CV events in this observational study given the RCT evidence of benefit in older adults [6]–[8]. Participants may have been less adherent to their anti-hypertensive regimen than in RCTs. Because medication intensity was measured based on prescriptions filled, however, nonadherence was probably not the major explanation. Previous studies of older adults have found a higher rate of CV events with greater blood pressure lowering [27]–[31]. This may be a particular problem for older adults with a greater burden of disease and disability, a group included in much higher numbers in the current study compared with RCTs. Another explanation is that individuals at greater risk of CV events are more likely to receive anti-hypertensive medications; we may not have eliminated confounding by indication completely. This possibility is suggested by the increased risk of hospitalization for heart failure among anti-hypertensive users versus nonusers. This finding may reflect the use of anti-hypertensives to treat heart failure rather than a lack of effect on CV prevention. Another possibility that must be considered is that individuals in the current study were at greater risk for other health outcomes than participants in the RCTs. These coexisting conditions and competing outcomes may limit the effect of treating a single condition such as hypertension. Studies of older adults with multiple conditions that do not account for competing risk may overestimate the benefit of anti-hypertensive and other treatments [23], [26].

Anti-hypertensive treatment was associated with a reduced rate of total mortality. While it is reasonable to assume that many of the deaths in participants with CV events were from CV causes, this is only speculative because we lacked data on cause of death. Previous studies report mixed results concerning the relationship between anti-hypertensive treatment and total mortality. HYVET also found a mortality benefit with anti-hypertensive treatment in older adults. Conversely, the Cochrane review reported a total mortality benefit for older adults less than 80 years old but not for those over age 80 years; the latter result was replicated in another meta-analysis of many of the same trials [6], [9]. Some investigators found increased mortality with aggressive anti-hypertensive treatment [32]–[34]. We cannot exclude differences in anti-hypertensive users and nonusers as an explanation for our finding. A combination of the indication bias noted above (individuals at higher risk for CV events are treated more aggressively) plus contraindication bias (sicker people may be less likely to receive or tolerate anti-hypertensives than healthier individuals) could explain the observed lack of effect of anti-hypertensives on CV events yet beneficial effect on mortality. This is unlikely the entire explanation because systematic differences for all but BMI and cognitive impairment were eliminated by matching. Furthermore, a greater mortality benefit was observed in those with, than without, CV events, suggesting this was not solely a ‘healthy user” effect. Thus it is possible that anti-hypertensives may not reduce the occurrence of CV events but may reduce the mortality associated with these events. This observation requires further investigation given the potential clinical importance.

Despite the lack of association with CV events overall, there was a 27% reduction in strokes with moderate anti-hypertensive intensity, similar to the benefit reported in RCTs.^6^ The small sample size may have precluded finding statistical significance. The beneficial effect of anti-hypertensive medication on stroke occurrence was not seen in the high intensity group. Previous studies, including RCTs, have found an inverse relationship between the maximum treatment allowed and the benefit of treatment in older adults, suggesting moderate blood pressure lowering may offer the optimal stroke prevention benefit [9], [27]–[28]. The ongoing SPRINT trial will address the effect of intensity of blood pressure lowering in older adults who meet study criteria [35].

This study has several strengths. The nationally representative cohort enhances the generalizability of results to the older adult population. The well-characterized cohort allowed us to account for medical, socio-demographic, functional, and other factors that affect both the propensity to receive anti-hypertensive medications and to experience the CV and mortality outcomes. The Medicare claims and Vital Status data allowed us to reliably identify the occurrence of CV outcomes and death. The anti-hypertensive intensity measure included both number and dose of medications. To account for biases and confounding inherent in observational studies, we both adjusted for propensity score and created a more homogeneous, propensity score-matched, subcohort [17], [21]. Results were similar in the propensity-matched and adjusted analyses, supporting validity of the results.

There were limitations in addition to lack of cause of death data and inadequate power for some analyses. We lacked information on blood pressure readings so were unable to relate blood pressure levels to anti-hypertensive intensity or the outcomes. Higher medication intensity may represent resistant or complicated hypertension [36], although the lower mortality in those with higher intensity suggests this is not the sole explanation. Study results need to be corroborated in a large dataset of representative older adults in which blood pressure readings are available.

Inception cohorts are recommended as one means of limiting bias in observational studies and assuring that confounders are measured prior to initiation of medications [37], [38]. MCBS does not contain information on time of onset of hypertension or duration of anti-hypertensive treatment. Regardless, an inception cohort may not be appropriate for the current study because older hypertensive adults have had hypertension, and been on treatment, for many years. The clinical question for older adults is usually not whether to start treatment but rather what is the likely benefit of continuing treatment. Despite methodological challenges, prevalent users, therefore, do represent the patient population for whom the decision of whether to continue anti-hypertensive medications is relevant. Innate to observational studies, despite adjustment for a wide array of confounding factors, we cannot exclude the possibility of unmeasured confounders and that those who do not take anti-hypertensive medications may inherently be different from those who do.

Results from this study are not conclusive but do raise the possibility that all older adults may not accrue the magnitude of cardiovascular benefit from anti-hypertensive treatment suggested by RCTs. While no single study is sufficient to answer a clinical question, current findings challenge the assumption that results from healthy older adults extrapolate to all older adults.

Determining the amount of benefit likely to accrue from treatment of individual conditions and ensuring that benefits outweigh harms is particularly important for older adults with multiple conditions. On the one hand, results of this study suggest possible survival benefits of anti-hypertensives. On the other hand, there was less evidence of CV prevention than observed in healthy samples of older adults. Recent studies report modest evidence of increased risk of falls and serious fall injuries such as hip fracture with anti-hypertensives among older adults, suggesting that anti-hypertensive treatment is not without harm [39], [40]. The biases inherent in observational studies and the inappropriateness of extrapolating RCT results from healthy older adults support the need for an RCT to determine the benefit versus harm of anti-hypertensives in clinically representative older adults with multiple chronic conditions. While such a trial will be expensive and challenging, the clinical implications are enormous. Pending the results of further research, we cannot assume benefit in all older adults. Expert consensus recommends that potential benefits as well as harms of anti-hypertensives should be weighed carefully in older adults with multiple conditions [5], [41].
